# Role of cytokines in combinatorial immunotherapeutics of non‐small cell lung cancer through systems perspective

**DOI:** 10.1002/cam4.2112

**Published:** 2019-04-17

**Authors:** Pragya Misra, Shailza Singh

**Affiliations:** ^1^ National Centre for Cell Science SP Pune University Campus Pune India

**Keywords:** EGFR, IL‐1RB, MAPK, NF‐kB, NSCLC, systems biology

## Abstract

Lung cancer is the leading cause of deaths related to cancer and accounts for more than a million deaths per year. Various new strategies have been developed and adapted for treatment; still the survival for 5 years is just 16% in patients with non‐small cell lung cancer (NSCLC). Most of these strategies to combat NSCLC whether it is a drug molecule or immunotherapy/vaccine candidate require a big cost and time. Integration of computational modeling with systems biology has opened new avenues for understanding complex cancer biology. Resolving the complex interactions of various pathways and their crosstalk leading to oncogenic changes could identify new therapeutic targets with lesser cost and time. Herein, this review provides an overview of various aspects of NSCLC along with available strategies for its cure concluding with our insight into how systems approach could serve as a therapeutic intervention dissecting the immunologic parameters and cross talk between various pathways involved.

## 1 INTRODUCTION

Lung cancer accounts for major proportion of cancer cases worldwide, with an estimated 1.6 million deaths each year.^1^ Non‐small cell lung cancer (NSCLC) constitutes to be a group of histological subtype effecting 85% of total lung cancer patients of which lung adenocarcinoma (LUAD) and lung squamous cell carcinoma (LUSC) are the most common subtypes (National Cancer Institute SEER Cancer Statistics Review 2011). Although advancements have been made in early diagnosis and treatment, still majority of cases are diagnosed at a later stage with poor prognosis. Though smoking is the major etiology for most forms of lung cancer, LUAD is more common in never smokers’ specifically in women and in East Asia (American Cancer Society Facts and Figures 2015). These patients have been associated with environmental factors like pollution, exposure to carcinogens along with genetic susceptibility.^2^

Tobacco prevention strategies, in spite of being an important component to control lung cancer, are alone not sufficient prevention strategy to combat the disease. Newer therapeutic strategies need to be evolved and implemented for meaningful outcomes. However, substantial progress has been made in the last two decades with the development of targeted therapies and immunotherapies, still the challenge remains in identifying molecular origin for disease including identification of new genetic alterations and understanding the mechanism of resistance to targeted therapy.^3^ Better understanding of these aspects would allow for better responses to immunotherapy and provide rationale for design of newer drugs for combinatorial therapy. In this review, we provide an overview on recent progress in treatment strategies of lung cancer along with a conclusive focus on systems biology as an innovative tool to be exploited for treatment of the disease.

## 2 TYPES OF LUNG CANCER

Lung cancer is basically classified into two types: small cell lung carcinoma (15% of total cases) and NSCLC (85% of total cases).^4^ Non‐small cell lung carcinoma can further be subdivided into three histological subtypes: squamous cell carcinoma, adenocarcinoma, and large‐cell lung cancer. Adenocarcinoma accounts for almost 40% cases and is most common form of NSCLC, found in both smokers and nonsmokers, and is not gender specific.^5^ It is usually found in outer part of lungs,^6^ type II alveolar cells secreting mucus being its origin.^7^ Squamous cell carcinoma occurs in flat squamous cells which line the inside of the airways and in center of lungs. Its prevalence is around 25%‐30% and is strongly associated with cigarette smoking.^8^ Large cell (undifferentiated) carcinoma accounts for nearly 10%‐15% of lung cancer cases and can appear in any part of lung spreading very fast.

## 3 PATHOLOGY AND DIAGNOSIS OF LUNG CANCER

**Figure 1:**
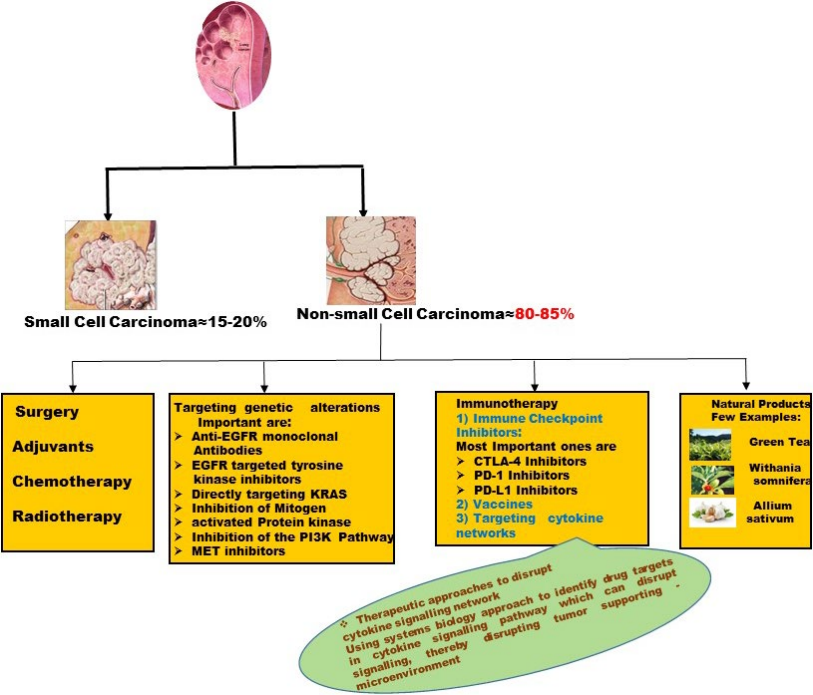
Non‐small cell lung cancer (NSCLC)—an overview

The World health Organization has established pathological diagnosis criteria's for lung cancer.^9^ The observation of clear morphological features of adenocarcinoma or squamous cell carcinoma in tissue samples obtained by bronchoscopy or surgical biopsy firmly establishes the diagnosis. Tumor is classified as NSCLC not otherwise specified, when no clear morphological evidence is found. Such tumors are further subdivided on basis of various other parameters such as mucin staining, various markers analyzed by molecular data and immunohisto/cytochemistry.^9^, ^10^ Various new marker genetic alterations are recommended now in the panel of molecular testing to classify NSCLC including mutations in epidermal growth factor receptor (EGFR), B‐Raf proto‐oncogene (BRAF), and the expression of programmed death ligand 1 (PD‐L1) in small biopsy samples and cytologic specimens 11, 12, 13, 14 (Figure 1).

## 4 GENETIC ALTERATIONS IN NSCLC

Molecular biology of NSCLC has been explored extensively in the last few years. Aided by high‐throughput techniques such as sequencing and genome analysis, these studies have identified various molecular alterations/events characteristic of NSCLC, which might be responsive to targeted therapy. This section summarizes the above said as exploiting these alterations/mutations for targeted therapy has been the first strategy for molecular‐guided therapy.

### 4.1 EGFR family

Epidermal growth factor receptor gene is a tyrosine kinase belonging to ErbB family and along with its ligand has shown various abnormalities in NSCLC including protein overexpression, gene amplification, and mutations leading to its progression.^15^, ^16^ The anomalous activities of EGFR along with helping in tumor growth and development also regulate various cellular activities like apoptosis and angiogenesis. Several groups have identified somatic mutations in EGFR in patients with lung carcinoma with increased frequency in patients who are nonsmokers, female patients, and patients from East Asian parts.^17^ Nearly 90% of these mutations are present in first four exons (18‐21) of tyrosine kinase domain of the EGFR gene, which are either an in‐frame deletion in exon 19 or a missense mutation in exon 21.^18^, ^19^, ^20^, ^21^, ^22^ Other tyrosine kinases involved in resistance mechanism include insulin‐like growth factor 1 receptor, *KRAS *mutations, and the epithelial‐to‐mesenchymal transition.^23^

Human epidermal growth factor receptor 2 (HER2), also known as NEU, EGFR2, or ERBB2, is another member of EGFR family.^23^, ^24^, ^25^, ^26^, ^27^ Mutations in HER2 have been identified in LUAD patients^28^ however, the frequency of such mutations is less than 5%. All the HER2 mutations were found in exon 20 and were in frame insertion mutations. These mutations are found more in nonsmokers and females. Among other members of this family, HER3 kinase mutation was not found in patients with NSCLC whereas HER4 kinase domain mutation was present in 2%‐3% Asian patients and was associated with smoking.

### 4.2 RAS mutation

RAS genes comprise of a family of GTP‐binding proteins which are membrane bound and regulate cell growth, differentiation, and apoptosis. Investigators have found that lung cancer patients frequently have somatic mutations in KRAS. RAS mutations usually occur as point mutation in the gene when an amino acid at position 12, 13, or 61 is replaced. It has been found that about 15%‐30% of LUAD has mutations in KRAS, a member of RAS family and is the reason for resistance to EGFR inhibitors (tyrosine kinase inhibitors and cetuximab) and chemotherapy.^29^ Most of these mutations are transversion mutations which effect exon 12 in 90% of patients and rest in exon 13. Rare coexistence of EGFR and KRAS mutations have been found in same tumors indicating that at functional level both the mutations have comparable impact in tumor progression.^23^, ^30^, ^31^, ^32^ These mutations have a very little prognostic significance. To further ascertain the role of these mutations in lung cancer, a transgenic mouse model was developed involving KRAS mutation and it was found that mice having these mutations are more susceptible to range of tumor types, specifically early growth of lung cancer.^33^ Other mutations found in patients of NSCLC include BRAF mutations which can have early occurrence in lung tumorigenesis.^34^

### 4.3 PI3K/Akt/mTOR

It is well proven that PI3K/AKT/mTOR signaling is activated in NSCLC and has importance in lung carcinogenesis. Studies have shown that phosphorylation of AKT and downregulation of PTEN, the negative regulator of this pathway occurs in NSCLC and is related to poor prognosis.^35^, ^36^ Downstream activation of this pathway also contributes in resistance to EGF receptor tyrosine kinase inhibitors. It has further been observed that amplification of mesenchymal‐epithelial transition (MET), one of the resistance mechanism involved in resistance to EGFR tyrosine kinase inhibitor can activate PI3K/AKT/mTOR pathway and inhibitors of PI3K pathway could overcome EGFR TKI resistance. Promising data has been observed with targeted agents against this pathway in early clinical trials for lung cancer management. Inhibitors have been designed for this pathway under various categories which include Pan‐PI3K inhibitors binding to the catalytic p110 subunits of class IA PI3Ks, PI3Kα, PI3Kβ, PI3Kδ, and PI3Kγ. GDC 0941 is the first oral Pan‐PI3K inhibitor under phase 1 study tested alone and in combination.^37^

## 5 IMMUNOLOGY OF NSCLC AND CELLS INVOLVED IN IMMUNE RESPONSE/CELL‐SPECIFIC IMMUNE RESPONSE IN NSCLC

Both infectious and non‐infectious foreign materials elicit an immune response along with infectious diseases, The immune system plays a major role in defense against cancer cells as well. Exploiting the immune milieu to identify new therapeutic strategies would be a better alternative for ongoing treatment strategies. Herein, this section we have tried to summarize role of cells of innate and adaptive immune system in NSCLC:

### 5.1 Role of CD4+ T helper cells

Immune and inflammatory responses are modulated through secretion of cytokines by various population of T helper cells after their activation. A very crucial role of CD4+ T lymphocytes in development of tumor has been well established. Th1/Th2 cell ratio in the peripheral blood of NSCLC patient serves as a prognostic marker for the disease.^38^ Data shows that patients with low ratio have an increased 5‐year survival by nearly 25% vs patients with a high ratio.^39^ Now, the role of IL‐17 has also been studied in case of NSCLC which is considered to be an important cytokine in tissue inflammation and immune promotion.^40^, ^41^, ^42^ There are controversial reports regarding role of IL‐17 in NSCLC; on one hand, few reports suggest its role in tumor cell proliferation and angiogenesis.^43^, ^44^ On the other hand, few reports have shown IL‐17 to induce tumor eradication.

### 5.2 Role of Th1 and Th2 cells in NSCLC

Proinflammatory cytokines secreted by Th1 cells have a detrimental effect on tumor leading to tumor rejection and antitumor progression.^46^ Pancreatic β‐cell cancer mouse model has been used to demonstrate the role of these cytokines on tumor, wherein the group has shown the combined effect of interferon‐γ (IFN‐γ) and TNF mediated through arrest of STAT1 and TNFR1 signaling along with p16INK4a leading to senescence of Tag‐expressing cancers. IFN‐γ plays a major in protection from tumor metastasis.^47^ In case of myeloma and B‐cell lymphoma, IFN‐γ induces macrophages for their direct cytotoxic effect against cancer as well as secretion of angiostatic chemokines.^48^ The importance of TNF‐α, a crucial Th1 cytokine, in tumors has been established using a TNF‐α knockout in a cancer mouse model showing early tumor development. This suggests that TNF plays a critical role in immune response against tumors.^49^ However, TNF‐α has been considered as a cytokine with dual role in cancer progression with some recent evidences, showing its pro‐tumorigenic effects. High serum levels of TNF‐α are found in patients of NSCLC with possibly a positive prognostic value. CD8+ cytotoxic T lymphocytes (CTL) activation and proliferation is induced by Th1 cells specifically against cancer cells. It has been shown that concomitant of high CD8+ T‐cell and high CD4+ T‐cell infiltration increases the survival rate in NSCLC patients.^50^ Th2 cytokines have an immunosuppressive role leading to tumor progression. It has been shown both in vitro and in situ that cells of human NSCLC produce Th2 cytokines. IL‐4 promotes lung cancer growth by inducing protease activity of cathepsin in macrophages associated with tumor.^51^, ^52^ Similarly, IL‐6 also serves as protumorigenic cytokine promoting STAT‐3 and NK‐kB pathways which help in activating survival and anti‐apoptotic signaling.^53^ IL‐10 has also been found to have role in aggressive tumor growth and there expression by NSCLC cells leads to significantly poorer prognosis.^54^ Along with these cytokines, IL‐13 also promotes growth and survival of tumor cells, thereby suppressing cell‐mediated immunity.^55^, ^56^

### 5.3 The Th17 and Treg paradigm

#### 5.3.1 Th17 cells which serve as a source of IL‐17 cytokine constitutes another Th subset which have role in autoimmunity and tumor

It has been reported that Th17 cells shows antitumor effect by recruiting and activating effector immune cells.^57^ The population of Th17 cells which differentiates in the presence of IL‐6, IL‐1β, and IL‐23, expresses high levels of IL‐2, IL‐33, and IL‐18r1, coexpression of RORC (RAR‐related orphan receptor C) and T‐bet, and significantly enhanced the ability to produce IFN‐γ.^58^ Synergistic role of IL‐17 produced by these cells along with IFN‐γ stimulates recruitment of tumor‐infiltrating effector T cells. Improvement in survival of lung cancer patients with high number of Th17 cells in pleural effusion was observed.^59^ In contrast to this, it has been observed that the high expression of IL‐17 leads to tumor growth by upregulating various survival‐associated genes and activation of NF‐κB signaling pathway.^60^, ^61^, ^62^

High Treg cell number in tumor tissue samples and peripheral blood of NSCLC patients are found to promote the tumor growth. It was found that COX‐2/PGE2 from tumor cells induces the activation of Treg cells with Foxp3 expression.^63^, ^64^ The overexpression of TGF‐β in both SCLC and NSCLC correlates with disease stage. Low levels of TGF‐β synergize with IL‐6 and IL‐21 promoting Th17 differentiation and its high levels promote Treg response.^65^, ^66^

### 5.4 Macrophages

Macrophages present in lung tumors are M2 macrophages secreting anti‐inflammatory cytokines IL‐10 and TGF‐β, thereby promoting metastasis and angiogenesis.^67^, ^68^ These macrophages also produce mediators such as VEGF and COX‐PGE2 which also promotes tumor growth. M1 subset of macrophages have been positively co‐related with NSCLC as they inhibit tumor growth by secretion of pro‐inflammatory cytokines like IFNγ, expression of inducible nitric oxide synthase, major histocompatibility complex (MHC) molecules, and reactive oxygen and nitrogen intermediates.^69^

### 5.5 Dendritic cells

A positive correlation has been established between number of mature DCs and the survival time in a study of tumor‐infiltrating immune cells of NSCLC patients (in an univariate analysis but not in a multivariate analysis, which calls for caution in using DC number to predict patient outcome).^70^ A surgical biopsy specimen in NSCLC patients showed varying population of DCs. CDc11 high myeloid DCs were semi‐mature expressing a higher, but limited, level of five markers chosen to indicate DC maturity (CD80/B7‐1, CD86/B7‐2, the DC activation marker CD83, HLA‐DR, and CD208/DC‐LAMP). The isolated CD11c‐plasmacytoid DCs were immature. Along with this, a third population was observed showing low levels of co‐stimulatory molecules and high levels of the immunoinhibitory molecule B7‐H1.^71^ More rigorous insight is required to further elucidate the underlying mechanism and clinical significance of these cancer‐associated DC subpopulations for their therapeutic anticancer function.

### 5.6 Natural killer cells

Natural killer (NK) cells are cytotoxic innate cells which have a vital role in the cytokine network of immune system. The activation of NK cells by cytokines such as type I interferons, IL‐12, and IL‐18 releases cytolytic granules for targeted cell disruption and cytokines for further immune response.^72^ NK cells primarily release IFN‐α, Th2‐associated cytokines, such as IL‐5 and IL‐13, and the regulatory IL‐10 cytokine may be released.^73^ Studies have shown that NSCLC cells release such soluble factors which inhibit the expression of granzyme B and IFN‐γ in intratumoral NK cells. It has been found that tumor‐infiltrating NK cells show proangiogenic activity, with production of VEGF, placental growth factor, and IL‐8/CXCL8.^74^, ^75^ The high frequency of Treg and low frequency of NK cells have been observed in the malignant areas with vice versa situation in nonmalignant areas demonstrating strong cytolytic activity ex vivo.^76^, ^77^, ^78^ Their role in both adaptive and innate immunity makes NK cells an attractive target for therapeutic development.

### 5.7 CD8+ cytotoxic T lymphocytes

CD8+ T cells have a very important role in cancer immunology due to their ability to recognize and destroy cancer cells.^79^ However, immunosuppressive mechanism of tumor cells impairs T cells for its survival. An increased Treg cell frequency also contributes to immunosuppression thereby escaping tumor cells from antitumor immunity. High CTL in tumor‐infiltrating cells indicate positive prognosis. Higher Treg/CTL frequency indicates poor response to therapy in NSCLC.^80^ Antitumor role of CTLs makes it a primary target for immunotherapeutic strategies against NSCLC since tumor‐specific CTLs show high expression of PD‐1 and become anergic. Along with anti‐PD‐1 therapy, development of adoptive T‐cell therapy and genetically engineered T cells which utilize chimeric antigen receptor technology, consisting of a junction between antibody components at the membrane surface and intracellular tails, to induce T‐cell proliferation and activity, thereby allowing MHC independence in T‐cell targeting is also in progress.^81^

## 6 THERAPEUTIC STRATEGIES AGAINST NSCLC WITH SPECIAL FOCUS ON IMMUNOTHERAPEUTICS

There has been a great advancement from cytotoxic therapies to targeted therapies in treatment for NSCLC in the last two decades. In this section, we have tried to summarize the ongoing therapies including immunotherapy, natural products as immunomodulators in NSCLC with a conclusive glimpse on incorporating systems biology approach in NSCLC treatment.

### 6.1 Adjuvant chemotherapy

Resection surgery is still the basic treatment for the patients with localized NSCLC.^82^ There is usually a substantial risk of relapse even after complete resection. Adjuvant therapy either in the form of radiation, chemotherapy, or targeted therapy reduces these risks. Adjuvant chemotherapy (AC) has slightly increased (4%‐5%) the 5‐year survival according to published meta‐analysis. Currently, patients having II and III pathological stages which showed curative intent postsurgery are recommended for AC 83).

### 6.2 Therapy based on targetable gene alterations

Tumor genotyping for identification of genetic alterations has helped in deciding the targeted therapy individualized for the patients.^84^, ^85^, ^86^ Usually mutations observed in tumors occur in the genes encoding proteins of signaling pathways which are involved in cellular proliferation and survival. These mutations help in formation and maintenance of tumors. Maintenance of the malignant phenotype of cancer cells is often physiologically dependent on the continued activity of specific activated, mutated or overexpressed oncogenes, phenomenon termed as “oncogene addiction.” This notion of oncogene addiction that tumors have genetic lesions which could be systematically identified helped in identifying new cancer drug targets. We have discussed above about various genetic mutations and there role in NSCLC. In this section, we are discussing some of the important therapies being targeted for these mutated genes.

## 7 EGFR

### 7.1 EGFR targeted therapies

We have discussed the role and importance of EGFR in NSCLC. Basically, two therapeutic approaches are being developed for target EGFR which include: (a) monoclonal antibodies against EGFR which bind to extracellular domain and (b) small molecule tyrosine kinase inhibitors targeting intracellular TK domain. Recently, anticancer role of various chemopreventive agents in NSCLC has been explored in downregulating EGFR at the gene level.

### 7.2 Anti‐EGFR monoclonal antibodies

Anti‐EGFR monoclonal antibodies are designed against the extracellular domain of EGFR and bind to it in its inactive state. This binding is competitive binding thereby obstructing the interaction of EGFR with its ligand and results in blocking of its activation and downstream signaling. These antibodies are very specific and exclusively bind to EGFR.

These monoclonal antibodies not only competitively inhibit EGFR binding but also induce receptor internalization and downregulates surface EGF receptors in a dose‐dependent manner.^87^, ^88^ The other mechanisms include antibody‐dependent cell‐mediated cytotoxicity and complement‐mediated cytotoxicity. The available anti‐EGFRmAbs include cetuximab, necitumumab, panitumumab, and matuzumab.

#### 7.2.1 Cetuximab

Cetuximab also known as C225, ErbituxTM, is a chimeric monoclonal antibody (human‐murine chimera) and it binds with many folds higher affinity with EGFR as compared to its natural counterpart. It was approved in 2004 by FDA and by Committee for Medicinal Products for Human Use in 2008 for combination therapy along with platinum‐based therapy in patients of head and neck squamous cell carcinoma having metastatic stage. It was also approved along with radiation therapy for locally advanced cancer.^89^ Cetuximab has been assessed as an addition to two phase III clinical trials in patients with NSCLC. Three patients in FLEX trial^56^ were randomly treated with cisplastin and vinorelbine along with or without cetuximab. It was observed that a marginal increase in the median overall survival of 11.3 months was seen with cetuximab when compared to 10.1 months with chemotherapy alone. However, a phase II trial, BMS099 (Pages et al, 2010) in which patients with NSCLC were treated with platinum‐taxane chemotherapy along with or without cetuximab showed no better survival. The reason for differences in results of both the trials could not be explained. Other EGFR inhibitors include Necitumumab, a monoclonal antibody against EGFR, which has undergone phase III trial and showed an improved overall survival (ClinicalTrials.govidentifier: NCT00981058). Two monoclonal antibodies, namely panitumumab (ClinicalTrials.gov identifiers: NCT01038037; NCT01088620) and matuzumab (ClinicalTrials.gov identifier: NCT00111839) are under phase II trial (Table 1).

**Table 1:** Targeted therapy

Target gene	Molecular feature	Drug approved/under study	Dose	Status	Diagnostic test
EGFR	Deletion in exon 19 and exon 21 activating mutations	Erlotinib	150 mg/mL orally (new dose: 100 mg/mL followed by 50 mg/mL)	Approved	PCR, next gen sequencing
		Gefitinib	250 mg/day orally	Approved	
		Afatinib	40 mg/day orally (new dose: 30 mg/day followed by 20 mg/day	Approved	
		Osimertinib	80 mg/day orally (new dose: 40 mg/day)	Approved	
KRAS	Mutation in codon 12 (90%), codon 13 (10%)	Selumetinib Trametinib Abemaciclib		None approved	PCR, next gen sequencing
BRAF	Mutations of tryrosine kinase domain, mainly Val600Glu mutations	Dabrafenib Vemurafenib Trametinib		None approved	PCR, next gen sequencing
HER2	Activating exon 20 insertions	Dacomitinib Transtuzumab T‐DM1		None approved	PCR, Next Gen Sequencing
MET	Exon 14 skipping mutations	Crizotinib Cabozantinib Capmatinib		None	PCR, Next Gen Sequencing
ALK	Fusion of partner gene with exon 20 of ALK; partners in fusion are EML4, KIF5B,TFG and KLC1	Crizotinib		Approved	
		Ceritinib		Approved	
		Alectinib		Approved	
		Brigatinib PF06463922 Other ALK inhibitors		None approved	
ROS1	Nine fusion proteins described with the FIG, SCL3442,TPM3,SDC4,EZR,LRIG3,KDELR2,CCDC6 genes	Crizotinib Ceritinib Cabozantinib		None approved	FISH, Immunohistochemistry
RET	Fusions described with four Partner genes:KIF5B,CCDC6,NCOA4,TRIM33	Vandetanib Cabozantinib		None approved	FISH
NTRK	Fusion of NTRK1 and NTRK2 occur with a range of partners	Entrectinib LOXO‐101		None approved	FISH

### 7.3 Tyrosine kinase inhibitors targeting EGFR

Inhibitors designed against EGFR are small molecules which are adenosine triphosphate (ATP) analogs binding either reversibly or irreversibly. These small molecules which are designed have competitive binding with ATP binding pockets which are present on intracellular catalytic kinase domain of tyrosine kinases receptors. This binding prevents autophosphorylation and downstream signaling of EGFR.^90^

Reversible inhibitors designed compete with ATP molecules to recognize the kinase active conformation. Binding of irreversible inhibitors on the other hand is a covalent interaction with nucleophilic cysteine residues at the active site of kinases. EGFR inhibitors are classified into three generations. Reversible inhibitors comprise the first generation. Patients with activating *EGFR *mutations (L858R and Del19) have shown good response with these inhibitors. The major drawback with these inhibitors was development of tumor resistance mainly due to *EGFR *T790M resistance mutation after a period of time.

#### 7.3.1 First‐generation inhibitors

##### Gefitinib—(ZD1839/Iressa)

Gefitinib, characterized in 1996, is an anilinoquinazoline derived orally active selective tyrosine kinase inhibitor with no inhibition of serine‐threonine kinase activity.^91^, ^92^, ^93^ This inhibitor has been approved for patients with NSCLC after failure of standard therapies and also in combination to standard cytotoxic drugs where it has shown dose‐dependent increase in growth inhibition.^94^ Its mode of action is not completely clear, however, it is found that it upregulates cyclin‐dependent kinase (CDK) inhibitor p27 and downregulates transcription factor c‐fos, resulting in the inhibition of CDK activity and G1 phase cell cycle arrest^95^ (Table 1).

##### Erlotinib—(OSI‐774; Tarceva)

Another orally active potent reversible inhibitor of EGFR is Erlotinib hydrochloride. Its mechanism also includes competitive binding in ATP binding pockets of receptor tyrosine kinases. Erlotinib induces apoptosis and cell cycle arrest in the G1 phase.^96^, ^97^ This inhibitor is used in NSCLC patients with relapse cases and advanced stage NSCLC patients showing stable diseased stage post‐treatment with four cycles of platinum‐based first‐line chemotherapy (Table 1).

#### 7.3.2 Second generation *EGFR *inhibitors

It was observed that resistance developed in patients treated with first‐generation inhibitors. Understanding the mechanism of resistance in patients post‐treatment with gefitinib, erlotinib, or afatinib, was the first step to unravel and identify alternative treatment strategy. It was found that T790M is the main resistance mechanism involved in such cases. This finding led to development of many drugs which target T790M. Drugs developed based on this strategy (neratinib, afatinib, and dacomitinib) demonstrated considerably good activity against T790M activity in the laboratory; however, the results of clinical studies in NSCLC patients were poor with response rate of less than 10% in the patients who have developed resistant to gefitinib or erlotinib.^98^, ^99^ In spite of so many inhibitors in pipeline, no second‐generation agents have been found to show effective response^100^, ^101^ (Table 1).

#### 7.3.3 Third‐generation *EGFR* inhibitors

A lot of third‐generation *EGFR *inhibiting molecules have been designed and developed actively to target T790M specifically and efficiently. The first inhibitor to receive FDA and EMA approval in November 2015 and February 2016, respectively, for NSCLC patients showing T790M was osimertinib (AZD9291) (Table 1).

##### Osimertinib (AZD9291; Tagrisso®)

Osimertinib has a different structure from other first‐ and second‐generation EGFR inhibitors. This molecule is a mono‐anilino‐pyrimidine compound binding covalently and has shown promising activity against various EGFR mutations like L858R, L858R/T790M, exon 19 deletion, and exon 19 deletion/T790MCross DAE^102^ (Table 1).

##### Olmutinib (BI‐1482694/HM61713; Olita^™^)

Olmutinib (Olita^™^) is an oral small‐molecule tyrosine kinase inhibitor of EGFR developed by Boehringer Ingelheim and Hanmi Pharmaceutical Co. Ltd. It binds covalently with receptor and leading to irreversible enzymatic inhibition of activating EGFR mutations and T790M mutation; however, it does not affect/bind with wild‐type EGFR. This molecule got the designation of “breakthrough therapy” in NSCLC by FDA in 2015. To evaluate its safety, tolerability, pharmacokinetics, and preliminary activity, a phase I/II trial HM‐EMSI‐101 (NCT01588145) 103 was conducted in patients of NSCLC pretreated with EGFR TKI in Korean population (patients were treated with 75‐1 200 mg/day of olmutinib). The overall response rate of 58.8% was observed in patients treated with dose more than 650 mg. In phase II trial, 76 patients of NSCLC who were confirmed for T790M were treated with 800 mg daily dose and the overall response rate was found to be 61%. In spite of encouraging clinical data, the development of this drug was stopped by Boehringer Ingelheim due to an unexpected increase in grade 3/4 skin toxicity (epidermolysis).

##### Nazartinib (EGF816)

This novel inhibitor specifically target L858R, Del19, and T790 M mutations.^104^, ^105^ The first human Phase I/II study [NCT02108964 (EGF816X2101)] with nazartinib includes 152 patients who were treated with this inhibitor once daily at doses ranging from 75 to 350 mg. The overall response rate and disease control rate were found to be 46% and *0.1%, respectively. Diarrhea, rashes, and pruritus were among most common toxic symptoms. This drug is also undergoing phase II trial in combination with nivolumab, an anti‐PD‐1 monoclonal antibody in *EGFR *mutant/T790M+ NSCLC patients who have progressed on first‐line *EGFR *TKI (NCT02323126) (Table 1).

## 8 KRAS

In the above sections, we have discussed the role of KRAS mutation in NSCLC. Mutations in KRAS gene are the most common molecular abnormalities in human malignancies. There are various strategies to target KRAS mutated NSCLC:

### 8.1 Directly targeting KRAS

Exceptionally high affinity to GTP/GDP along with the absence of allosteric binding sites, post‐translational modifications, and multiple compensatory pathways providing parallel signaling routes, make KRAS an attractive target.^106^ Blocking the post‐transcriptional addition of a farnesyl group to KRAS by using a farnesyl transferase inhibitors (FTI) was tried.^107^ However, this pathway has an alternative escape pathway through the activation of process of post‐transcriptional geranylgeranylation. The development of FTI as monotherapy was banned after two inhibitors namely R1155777 and salirasib, which is a farnesylcysteine mimetic, 108 were inactive in a KRAS‐mutated NSCLC cohort.^109^

Various molecules, SML‐8‐73‐1(targets the guanine nucleotide binding pocket of the KRAS product of the G12C mutation)^110^ and ARS‐853(binds to KRAS/G12C),^111^ have shown encouraging preclinical data. These data suggests that KRAS is a potent drug target (Table 1).

### 8.2 Inhibition of mitogen activated protein kinase

These kinases are downstream effectors in MAPK signaling cascade. It was thought that these could be suitable targets. MEK inhibitors showed modest efficacy in clinical trials. Selumetinib which is a selective allosteric inhibitor of MEK1/MEK2 showed good preclinical activity in KRAS‐mutated cancers.^112^ The combination of selumentinib and docetaxel when compared with docetaxel monotherapy showed better overall response ratio in a phase 2 trial; however, no change was observed in overall survival with increased chances of side effects which include febrile neutropenia (14%) for the combination versus 0% for docetaxel.^113^ Trametinib which is also a MEK inhibitor has received regulatory approval for v‐Raf murine sarcoma viral oncogene homolog B (BRAF) mutated advanced melanoma. Data suggest that in spite of a systemic rationale to target downstream molecules like MEK of KRAS mutation, clinical trials have not shown much exciting data (Table 1).

### 8.3 Inhibition of the PI3K pathway

It has been observed that mutations in PI3K catalytic subunit coexist with KRAS mutations and PI3K/AKT signaling increases in KRAS mutated cells. It was hypothesized from the preclinical data that inhibiting both MEK and PI3k can have a better effect. Based on this hypothesis, pretreated patients having various KRAS mutated tumors were taken for a phase I trial and were given AKT inhibitor in combination with selumetinib. Results were promising as 23% NSCLC patients showed objective response.^114^

Various other kinases have also been suggested and tested for targeted therapy due to their close association with KRAS. Cyclin‐dependent kinases 4 and 6 are important for KRAS‐driven oncogenesis and thus can be a putative target in NSCLC.^115^ Abemaciclib, a CDK4/6 inhibitor, showed promising data in patients with KRAS mutation. Encouraged by these data, a phase III trial (JUNIPER, NCT02152631) to compare abemaciclib with erlotinib has been started in NSCLC patients having KRAS mutations post‐treatment.^116^ Along with this, inhibitors for focal adhesion kinase (defactinib) have also shown positive results. Various other multikinase inhibitors in NSCLC are designed against the vascular endothelial growth factor receptor, platelet‐derived growth factor receptor, B‐Raf, and RAF proto‐oncogene serine/threonine‐protein kinase (c‐Raf) which serve as potential targets.^117^ Studies have also been undertaken to use HSP90 inhibitors, which is a molecular chaperone in NSCLC. However, not much encouraging data has been obtained using inhibitors against HSP90 (Table 1).

### 8.4 MET amplification in NSCLC

Hepatocyte growth factor receptor (HGFR), gene product of MET, serves as a potential drug target in NSCLC. MET is found to be amplified in about 5% of LUAD. There are evidences which suggest role of MET activation as a primary oncogenic driver and a secondary driver for resistance in targeted therapy. Strategies used to inhibit MET/HGFR pathway include antagonists of HGFR as well as monoclonal antibodies against HGFR and MET. Tyrosine kinase inhibitors against MET under study are tivantinib (ARQ197), cabozantinib (XL184) and crizotinib 118. Exploiting the synergistic role of MET and EGFR, dual inhibitors viz. erlotinib and trivantinib have been tested in non‐squamous NSCLC in the global phase III trial MARQUEE. However later data were not so promising to continue the study (Table 1).

## 9 IMMUNOTHERAPY IN NSCLC

Tumor cells develop the ability to escape the immune system by using certain inhibitory pathways and thereby disturb the immune checkpoint of host by various ways so as to avoid exclusion by the host immune system.

### 9.1 Immune checkpoints in cancer immunotherapy

Certain inhibitory pathways are there in the immune system to maintain self‐tolerance, which are used by tumors to escape immune surveillance.^119^ Tumor cells overexpress inhibitory ligand and receptors which regulate T‐cell effector functions as a survival strategy.^120^ Once these immune checkpoints are blocked, antigen‐specific T‐cell responses are restored. Among various checkpoint inhibitors, CD28/cytotoxic T‐lymphocyte antigen 4 (CTLA‐4) axis, and PD‐L1/PD‐1 have been explored a lot and they have been shown to serve as potent drug targets. Other than these two, various other molecules such as TIM3, B7H3, VISTA, LAG3, and TIGIT are also explored for their ability as drug targets for cancer immunotherapy 121, 122 (Table 1). This section reviews the major advances in exploiting these molecules as cancer immunotherapeutics.

## 10 CTLA‐4 INHIBITORS

CTLA‐4 expressed on T cells regulates its activation by counteracting the activity of T‐cell costimulatory receptor CD28. Various inhibitors have been designed which include:

### 10.1 Ipilimumab

It is a humanized anti‐CTLA‐4 monoclonal antibody which blocks CTLA‐4 binding to its ligand. Treatment‐naïve stage IV NSCLC patients were tested in a random phase II clinical trial for paclitaxel and carboplatin with or without ipilimumab. These patients showed an improvement in immune‐related progression‐free survival with ipilimumab, when ipilimumab was given after chemotherapy^123^ however, no significant increase was observed in overall survival. Ipilimumab was given after chemotherapy so that antigen could be released before immune modulation. Phase III trial for ipilimumab is still ongoing. Toxicities associated with this treatment included anemia, diarrhea, and fatigue; grade 3/4 immune‐mediated toxicities (colitis, transaminitis, and hypophysitis).

### 10.2 Tremelimumab

Tremelimumab was tested initially in advanced melanoma. This monoclonal antibody did not show any remarkable increase in survival of patients with metastatic melanoma when compared with standard chemotherapy in first‐line treatment.^124^ A recent clinical trial of tremelimumab along with anti‐PD‐L1 antibody is ongoing presently (NCT02000947). (Table 2).

**Table 2:** Immunotherapy

Status of immune checkpoint inhibitors				
Checkpoint inhibitor being targeted	Agent	Result	status	Side effect
CTLA‐4	Ipilimumab (Bristol‐Myers Squibb)	Improved overall survival in patients with previously treated metastatic melanoma	Approved for the treatment of unresectable or metastatic melanoma	Adverse events can be severe, long‐lasting, or both, but most are reversible with appropriate treatment
	Nivolumab plus ipilimumab, nivolumab plus chemotherapy, or chemotherapy.	Progression‐free survival was significantly longer with first‐line nivolumab plus ipilimumab than with chemotherapy among patients with NSCLC and a high tumor mutational burden	Phase III	Overall, treatment‐related deaths occurred in seven patients (1.2%) treated with nivolumab plus ipilimumab (three died from pneumonitis and one each died from myocarditis, acute tubular necrosis, circulatory collapse, and cardiac tamponade), six patients (1.1%) treated with chemotherapy (two died from sepsis and one each died from multiple brain infarctions, interstitial lung disease, thrombocytopenia, and febrile neutropenia with sepsis), and two patients (0.5%) treated with nivolumab (one each died from pneumonitis and neutropenia with sepsis)
	Tremelimumab (MedImmune/AstraZeneca)		Phase II	Diarrhea, skin rashes, dry and itchy skin, liver problems such as inflammation of the liver (hepatitis) and high levels of liver enzymes in your blood
PD‐1	Nivolumab (Bristol‐Myers Squibb) (Nature: human monoclonal IgG4 antibody lacking detectable antibody‐dependent cellular cytotoxicity (ADCC))	The drug demonstrated improved overall survival (OS) compared to docetaxel in patients with squamous and nonsquamous cell histologies progressing on platinum doublet chemotherapy in large Phase III trials	Phase III	Beside known adverse events like fatigue and diarrhea, severe toxicities with immunologic related events may also occur. It may also induce pneumonitis. Thyroid function abnormalities, in particular non‐autoimmune hypothyroidism and transient thyrotoxicosis on autoimmune basis, seem the major endocrine adverse event related to nivolumab
	Pembrolizumab (Nature: humanized monoclonal antibody having anti‐PD‐1 activity with mutation at C228P to prevent Fc‐mediated ADCC)	In NSCLC, a phase I clinical trials in patients with failed two systemic regimens showed an overall response rate of 24%. Other ongoing trials are pembrolizumab vs docetaxel	Phase II/III	The most common adverse reactions are fatigue, musculoskeletal pain, decreased appetite, pruritus, diarrhea, nausea, rash, pyrexia, cough, dyspnea, and constipation. Pembrolizumab is associated with immune‐mediated side effects, including pneumonitis, colitis, hepatitis, endocrinopathies, and nephritis
		Pembrolizumab vs platinumbased chemotherapy	Phase III	
		Single‐agent pembrolizumab vs. platinum‐based chemotherapy	Phase III	
		Single‐agent pembrolizumab	Phase II	
		Safety, tolerability, and efficacy of pembrolizumab 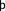 chemotherapy or immunotherapy	Phase I/II	
*On September 22, 2017, FDA approved pembrolizumab (KEYTRUDA, Merck & Co., Inc) for patients having metastatic, gastric, or gastroesophageal junction adenocarcinoma and positive for PD‐L1 expression as per an FDA‐approved test*				
PDL‐1	Atezolizumab (MPDL‐3280A) (Nature: human IgG1 antibody that targets PD‐L1)	Response rate of 21% was reported	Phase I trial	No serious side effect seen
		Further trials are evaluating MPDL‐3280A compared to docetaxel (NCT01903993 and NCT02008227)		
		MPDL‐3280A monotherapy in PD‐L1–positive NSCLC is ongoing (NCT01846416)	Phase II	
		Efficacy of combinatorial approaches with erlotinib (NCT02013219), bevacizumab (NCT01633970) and the MEK inhibitor cobimetinib (NCT01988896)	Phase I	
	On basis of these phase clinical trials, FDA granted atezolizumab *“a breakthrough therapy”* designation in February 2015 for the treatment of PDL1‐positive NSCLC Patients during or post after standard treatments. It is the first PD‐L1 inhibitor approved for use in patients with NSCLC who are on platinum‐doublet chemotherapy or appropriate targeted therapy			
	MEDI4736 (Nature: human IgG1 antibody specific for PD‐L1 binding)	Preliminary data from the NSCLC cohort of an ongoing phase I study in advanced solid tumors (NCT01693562) showed an overall survival rate of 13% at 12 weeks Further trials are evaluating: MEDI4736 vs. placebo after chemoradiotherapy	Phase III	No colitis or pneumonitis of any grade, with several durable remissions including NSCLC patients
		MEDI4736 post failure or >2 prior systemic treatment regimens	Phase II	
		Safety and tolerability of MEDI4736 and Tremelimumab	Phase Ib	
		Antitumor activity of MEDI4736 and gefitinib post failure of standard treatment	Phase I	

## 11 PD‐1 AND PD‐L1 INHIBITORS

### 11.1 PD‐1 inhibitors

PD‐1 is another important immune checkpoint, belonging to B7/CD28 family of receptors. Interaction of PD‐1 with its ligands namely PD‐L1 and PD‐L2 is responsible for regulating T‐cell activity. It inhibits T‐cell proliferation and function, thereby reducing the levels of IFN‐γ, tumor necrosis factor‐α, and IL‐2 production. This shows that high expression of PD‐1 indicates toward **“exhausted” **or** “anergic”** T cells. This state of T cells is unable to provide the cytokine milieu to control tumors. With a rationale that blocking these check‐points can restore T‐cell function, various anti‐PD‐1 antibodies have been developed and studied against NSCLC.

## 12 ANTI‐PD‐1 ANTIBODIES

### 12.1 Nivolumab

It is a human IgG4 monoclonal antibody which targets PD‐1. It has been observed since phase I clinical trial of monoclonal antibody against PD‐1, which showed activity against NSCLC, that blocking PD‐1 helps in restoration of T‐cell function, thereby leading to optimal cytokine secretion.^125^ A large phase I study with this antibody, which enrolled 296 patients out of which 236 were evaluated, showed that objective response of the recruited patients with NSCLC was 18%. Sixty‐five percent of the patients who responded showed a response lasting for more than a year. It was found that stable disease lasted for 24 weeks in patients of NSCLC. In another phase I trial with 129 pretreated patients of NSCLC, it was observed that objective response was shown by 22 patients and the median duration of this response was quite long for 17 months.^126^ Although the median overall survival was found to be 9.9 months, the patients who responded showed sustained benefit. Toxicities associated with this treatment included fatigue, low appetite, diarrhea along with pneumonitis reported by few patients. Recent ongoing phase III clinical trial is comparing the monotherapy of nivolimab with docetaxel as second‐line treatment (NCT01642004 and NCT01673867). Another phase III first‐line trial is recruiting NSCLC patients which are PD‐L1 positive for evaluating its efficacy as compared to standard chemotherapy (NCT02041533) (Table 1). Other anti‐PD1 antibody under clinical research trial includes *Pembrolizumab.* Its trial as monotherapy for NSCLC is ongoing (NCT01840579).^125^ Few randomized trials to compare it with combination chemotherapy (NCT02142738) or docetaxel (NCT01905657) have been initiated in patients with NSCLC positive for PD‐L1 (Table 2).

## 13 PD‐L1 INHIBITORS

Another major suppressor of antitumor activity is PD‐L1, ligand for PD‐1. It anergizes T cells by binding to PD‐1. A higher expression of PD‐L1 has been observed in many malignant cell population and studies have shown that blocking it with anti‐PDL‐1 antibody restores T‐cell function thereby leading to tumor suppression. Various antibodies have been developed and tested against PD‐L1 as follows:

### 13.1 BMS‐936559/MDX1105

It is a human monoclonal IgG4 antibody which binds with PD‐L1 thereby preventing the interaction of PD‐L1 with PD‐1.^127^ Results from a phase I trial which was multicentric with 207 patients, 75 patients of NSCLC showed tumor regression and prolonged stabilization of disease. Patients with NSCLC showed five objective responses with response rate of 8% and 16%, respectively, at doses of 3 mg/kg and 10 mg/kg.

### 13.2 MPDL3280A (Atezolizumab)

It is a human monoclonal IgG1 antibody against PD‐L1.^128^ It is the first PD‐L1 inhibitor to receive FDA approval for metastatic NSCLC patients who have received front line chemotherapy. Approval for this was based on data from two open‐label phase II multicenter trials, POPLAR (NCT01903993) and BIRCH (NCT02031458). Both these trials have shown the benefit in overall survival, progression‐free survival, and response rate in the patients treated with atezolizumab as compared to single‐agent docetaxol (Table 2).

## 14 THERAPEUTIC VACCINES

Therapeutic vaccines which include various strategies including recombinant tumor antigen proteins, peptides, tumor cells, primes the immune system to recognize tumor‐specific antigens and boost antitumor humoral and cellular immune response.^129^, ^130^ The renewed interest in therapeutic cancer vaccine has developed due to the in‐depth understanding of immune checkpoints in cancer and clinical success of immune checkpoint inhibitors along with advanced computational biology platform that enable the development of cancer neo antigen vaccination strategies.

Two most important vaccination strategies being used against NSCLC include whole cell vaccines and antigen‐specific vaccines.

### 14.1 Whole cell vaccines

#### 14.1.1 Belagenpumatucel‐L

It is an allogenic whole cell vaccine produced from irradiated four different cell lines of NSCLC transfected with antisense gene plasmid for TGF‐β2 to genetically modify it (Table 1). Along with antigenic diversity, this vaccine has antisense inhibition of TGF‐β2 expression, thereby increasing effector cell‐mediated antitumor response.^131^

### 14.2 Antigen‐specific vaccines

#### 14.2.1 Tecemotide (liposomal BLP25)

Tumor‐associated/ specific antigens can serve as a better vaccine candidate. Mucin1 (MUC1), a cell membrane glycoprotein is found to be overexpressed and aberrantly glycosylated in cancer.^132^ Tecemotide (L‐BLP25) is a MUC1 antigen‐specific peptide vaccine which has capacity to evoke a T‐cell response against this antigen which is overexpressing in NSCLC. This antigen has been evaluated for its efficacy in a phase III clinical trial for treatment of unresectable stage IIIA/IIIB NSCLC patients following chemotherapy.^133^

#### 14.2.2 Melanoma‐associated antigen 3

This contains complete recombinant protein (cancer/testis antigen33) which is formulated along with immunostimulant AS15. The expression of this protein has been found in 35%‐55% of NSCLC patients (stages I‐IV).^134^ In phase II clinical trials, the vaccine was not able to show progression‐free survival in stage IB/IIMAGE‐A3‐positive NSCLC patients 134, 135. In the MAGE‐A3 as Adjuvant Non‐Small Cell Lung Cancer Immunotherapy (MAGRIT) trial, patients which were enrolled were histologically confirmed to have resected stage IB, II, or IIIA MAGE‐A3 expressing NSCLC confirmed by polymerase chain reaction^136^. It was a random trial with patients treated with MAGE‐A3 and placebo‐treated control patients in ratio of 2:1. Patients treated with MAGE‐A3, received 13 intramuscular injections of this vaccine within the time frame of October 2007 to July 2012.

The median disease‐free survival for both the vaccine treated and placebo groups was 60.5 and 57.9 months, respectively. For the patients who have not received prior chemotherapy, it was 58.0 months in patients and 56.9 months in the placebo group indicating that this immunotherapy is not efficient in patient with surgically resected early NSCLC. This was not the alone negative vaccination trial; other two trials START trial and STOP trial also failed.

#### 14.2.3 TG4010

This vaccine is a combination of recombinant modified poxvirus which encodes MUC1 antigen along with IL‐2. This vaccine was tested as combination therapy with chemotherapy but did not show any significant effect in overall survival.^137^ A phase IIB/III trial for combination of this vaccine with first line of chemotherapy is ongoing (NCT01383148; Table 1).

### 14.3 Epidermal growth factor vaccine

Epidermal growth factor along with its receptor is overexpressed in various cancers including NSCLC. This pathway has been exploited a lot for designing inhibitors and has shown promising data. Vaccine with this protein CIMAVax was developed in Cuba which included recombinant human epidermal growth factor, P64K Neisseria meningitides carrier protein, and immunoadjuvant Montanide ISA 51. When administered, this vaccine generates antibody against EGF which inhibits binding of EGF to EGF receptor.^138^ A phase II trial conducted with patients having IIIB/IV NSCLC did not show any survival benefit for the patients, however the patients who developed antibody response showed a better overall survival 139 (Table 1).

## 15 NATURAL PRODUCTS: AN ATTRACTIVE APPROACH HAVING FEWER SIDE EFFECTS

Most of the available treatments for NSCLC mentioned above are associated with side effects such as drug resistance, toxicity in chemotherapy. Immunotherapy is also associated with various side effects as on‐target T‐cell toxicity against target antigens expressing in normal tissue, or breaking of self‐tolerance. Medicinal plants may serve as a rich repository for herbal medicines and phytochemicals against NSCLC. This treatment may serve as a safe and cost‐effective alternative treatment compared to conventional drugs as toxicity associated with medicinal plants is inconsequential. However, various medicinal plants are associated with severe toxicity. Therefore, the assessment of the toxicity of medicinal plants, as well as their herbal preparations, is essential to determine the applicability of the sample as a pharmacological drug. The major limitation with the usage of the medicinal plants in spite of many merits and gained popularity in the recent time is the insufficient information about their mechanism of action. Herein, we discuss in brief the medicinal plants in use against NSCLC:

### 15.1 Green alga *Chlorella vulgaris*, strain CK22

Various fractions of the green algae were tested for their antitumor activity against lung cancer. It was found that the most active fraction, Q2C2, which has about 56% of galactose‐rich carbohydrate and 36% protein has the maximum activity. This activity is lost after protease treatment indicating that it might be due to protein moiety of glycoprotein.^140^

### 15.2 Seaweed *Undaria pinnantifida*

Viva‐Natural, a natural product extracted from the dietary seaweed *Undaria pinnantifida *(Alariaceae), demonstrated therapeutic activity and showed an average efficacy when used prophylactically against LLC in allogeneic mice model. In vitro cytolytic activity of peritoneal macrophages against KB cells was increased posttreatment with this product suggesting that the Viva natural shows its antitumor response by activating nonspecific immune response.^141^ Compared to standard synthetic immunomodulators, the efficacy of Viva‐natural has found to be superior against LLC in some cases and inferior in others.^142^ It has shown synergistic or additive effect when given along with anticancer drugs.^141^

### 15.3 Garlic (*Allium sativum*)

It is a common plant used mainly as a food item, and is being used as a medicinal herb in various parts of world. A unique garlic preparation, called Aged Garlic Extract, show various tumor inhibitory effects. Its inhibited proliferation of LL/2 lung carcinoma (syngeneic) cells transplanted into mice has a significant immunostimulatory effect with antitumor activity mediated via increased activity of NK cell, macrophages, and reactivity of lymphocytes when stimulated with mitogens.^143^, ^144^

### 15.4 *Withania somnifera *(ashwagandha)

Numerous review articles about the chemical properties, therapeutic benefits, and toxicity of *Withania somnifera *(ashwagandha, WS), one of the most important herbs of Ayurveda (the traditional system of medicine in India), have been published. The antitumor activity was described in different in vitro and in vivo experimental models, including urethane induced lung adenoma in mice. The data showed that the ethanol extract of *W*.* somnifera *significantly reduced tumor incidence.^145^

### 15.5 Green tea

The primary catechins in green tea are epicatechin, epicatechin‐3‐gallate, epigallocatechin, and epigallocatechin‐3‐gallate. A clinical study investigated the chemopreventive effects of green tea and coffee among cigarette smokers. Data from this study indicates that polyphenols present in green tea may have an antimutagenic effect against smoke‐induced mutations in humans. It has also been shown that human lung cancer cell line (A549) when treated with polyphenols of green tea and later on exposed to smoke of cigarette solution or H_2_O_2_ had a reduced incidence of DNA strand breaks. These results suggest that green tea polyphenols may inhibit DNA damage and other mutations in cells exposed to oxidants and that this effect is associated with anticarcinogenic properties.^146^, ^147^ A recent article published evidence from epidemiologic studies on cancer prevention by green tea.

These data suggests that there is a need for elaborated studies which would not only explore the immunomodulatory efficacy of these Indian medicinal plants but also dissect their mechanistic aspects of action so that these could serve as a cost‐effective and safe alternative for NSCLC treatment.

## 16 SYSTEMS BIOLOGY IN NSCLC

All the above strategies to combat NSCLC either by identifying effective drug molecule or development of immunotherapy are high on cost and is an extensive work which needs years to be finished. A computational method along with systems biology would serve as a cost‐effective and time saving strategy to integrate existing information to identify new candidate drugs for further study and identify new drug targets. A study by Xiang et al identified 38 irreversible EGFR‐T790M inhibitors using this strategy.^148^, ^149^ One more study developed a computational model based on chemical‐/protein‐chemical interaction and identified molecules with anti‐NSCLC activity.^150^

**Figure 2:**
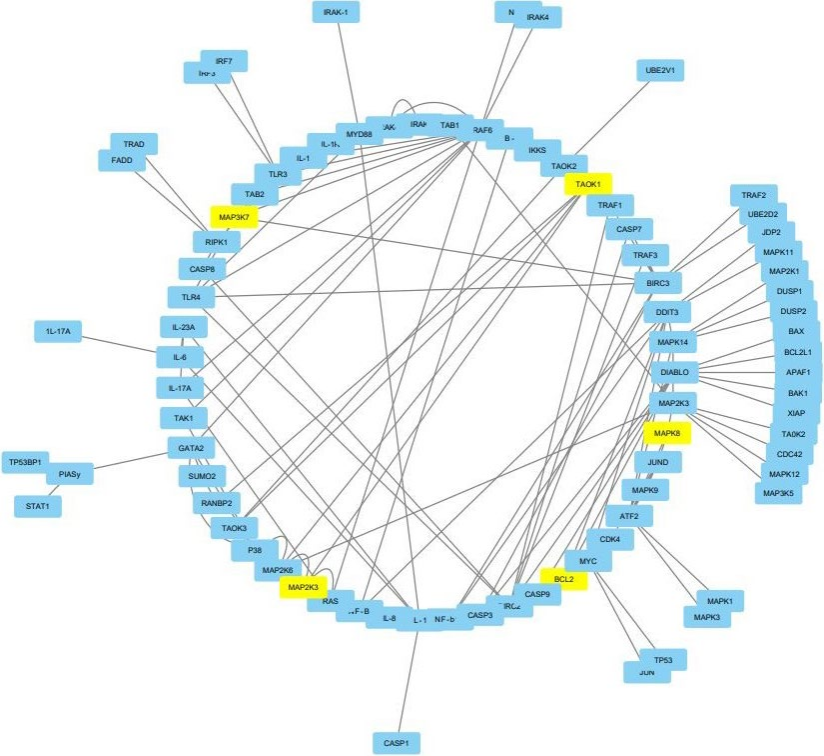
Reconstructed model of MAP2K3 signaling and NF‐kB

Integration of computational modeling with a plethora of theoretical experimental data has opened new avenues for cancer systems biology. The methodologies adopted for the same varies from network analysis to differential equation or correlative regression from an abstract model to a highly specified model. These molecular and cellular networks emphasize the systems aspect at the dynamic level. Transcription, translation, and posttranslational processes strongly influence the network biology throwing an insight into the associated components governing the behavior of the cell. How the components interact with each other to drive tumor progression is a major burning question in the field? The pathophysiological symptoms identified through the dysregulated pathways help discern the complex networks impacted by oncogenic changes in the human cells. Small perturbations in the network affects the feedback mechanisms whether be positive or negative. The model reconstructed herein Figure 2 highlights the cross talk examined between the MAP2K3 signaling and NFkB network indicated a role for TRAF6 in the synergistic view of the treatment of the disease. The network comprised of 84 signaling nodes and 126 interaction rules constructed via literature curation. Following refinement, the model was able to predict the conditions under which new inhibitors could be designed inhibiting IL‐1b receptors that would be sufficient to halt the cell cycle progression and also indicative of the fact that MYC could be suggested as a potential alternative therapeutic target. Explicit network topology and network parameters derived further suggest certain stochasticity which might be associated with cell‐to‐cell variation.

## CONFLICT OF INTEREST

The authors have no competing interest.

## AUTHOR CONTRIBUTIONS

PM and SS conceived the design concept and wrote the original draft. SS reviewed the draft and contributed in final preparation of the manuscript. Both authors approve the final publication of the manuscript.

## References

[1] Siegel R , Ma J , Zou Z . Jemal A . Cancer statistics. CA Cancer J Clin. 2014;64:9‐29. 24399786 10.3322/caac.21208

[2] Sun S , Schiller JH , Gazdar AF . Lung cancer in never smokers–a different disease. Nat Rev Cancer. 2007;7:778‐790. 17882278 10.1038/nrc2190

[3] Zhao X , Wang A , Walter V , et al. Combined targeted DNA sequencing in non‐small cell lung cancer (NSCLC) using UNCseq and NGScopy, and RNA sequencing using UNCqeR for the detection of genetic aberrations in NSCLC. PLoS One. 2015;10:e0129280. 26076459 10.1371/journal.pone.0129280 PMC4468211

[4] Sher T , Dy GK , Adjei AA . Small cell lung cancer. Mayo Clin Proc. 2008;83:355‐367. 18316005 10.4065/83.3.355

[5] Couraud S , Zalcman G , Milleron B , Morin F , Souquet PJ . Lung cancer in never smokers–a review. Eur J Cancer. 2012;48:1299‐1311. 22464348 10.1016/j.ejca.2012.03.007

[6] Travis WD . Surgical pathology of pulmonary infections. Semin Thorac Cardiovasc Surg. 1995;7:62‐69. 7612757

[7] Thunnissen E , Noguchi M , Aisner S , et al. Reproducibility of histopathological diagnosis in poorly differentiated NSCLC: an international multiobserver study. J Thorac Oncol. 2014;9:1354‐1362. 25122431 10.1097/JTO.0000000000000264

[8] Kenfield SA , Wei EK , Rosner BA , Glynn RJ , Stampfer MJ , Colditz GA . Burden of smoking on cause‐specific mortality: application to the Nurses' Health Study. Tob Control. 2010;19:248‐254. 20501499 10.1136/tc.2009.032839 PMC3050007

[9] Travis WD . Sarcomatoid neoplasms of the lung and pleura. Arch Pathol Lab Med. 2010;134:1645‐1658. 21043818 10.5858/2010-0086-RAR.1

[10] Travis WD . Advances in neuroendocrine lung tumors. Ann Oncol. 2010;21(Suppl 7):vii65‐vii71. 20943645 10.1093/annonc/mdq380

[11] Wu WS , Chen YM . Re‐treatment with EGFR‐TKIs in NSCLC patients who developed acquired resistance. J Pers Med. 2014;4:297‐310. 25563355 10.3390/jpm4030297 PMC4263962

[12] Wu X , Amos Ci , Zhu Y , et al. Telomere dysfunction: a potential cancer predisposition factor. J Natl Cancer Inst. 2003;95:1211‐1218. 12928346 10.1093/jnci/djg011

[13] Wu TH , Hsiue EH , Lee JH , Lin CC , Yang JC . New data on clinical decisions in NSCLC patients with uncommon EGFR mutations. Expert Rev Respir Med. 2017;11:51‐55. 27927060 10.1080/17476348.2017.1267569

[14] Wu X , Schabath MB , Spitz MR . Myeloperoxidase promoter region polymorphism and lung cancer risk. Methods Mol Med. 2003;75:121‐133. 12407737 10.1385/1-59259-324-0:121

[15] Sun G , Liu B , He J , Zhao X , Li B . Expression of EGFR is closely related to reduced 3‐year survival rate in Chinese female NSCLC. Med Sci Monit. 2015;21:2225‐2231. 26230859 10.12659/MSM.894786 PMC4554359

[16] Sato J , Horinouchi H , Goto Y , et al. Long‐term survival without surgery in NSCLC patients with synchronous brain oligometastasis: systemic chemotherapy revisited. J Thorac Dis. 2018;10:1696‐1702. 29707323 10.21037/jtd.2018.03.08 PMC5906248

[17] Kris MG , Tonato M . New approaches in the treatment of non‐small cell lung cancer: taxanes in the treatment of NSCLC: pathways to progress. Lung Cancer. 2002;38(Suppl 4):1‐3. 10.1016/s0169-5002(02)00166-6 12480188

[18] Jeong JH , Oh YJ , Kwon TK , Seo YH . Chalcone‐templated Hsp90 inhibitors and their effects on gefitinib resistance in non‐small cell lung cancer (NSCLC). Arch Pharm Res. 2017;40:96‐105. 27770383 10.1007/s12272-016-0848-z

[19] Mok T , Wu YL . Intercalated chemotherapy and erlotinib: a viable first‐line option for patients with advanced NSCLC?—authors' reply. Lancet Oncol. 2013;14:e438‐e439. 10.1016/S1470-2045(13)70434-0 24079868

[20] Zhuo Y , Guo Q , Song P , et al. Correlation study and significance of the EGFR expression in serum, lymph nodes and tumor tissue of NSCLC. Thorac Cancer. 2014;5:31‐37. 26766969 10.1111/1759-7714.12048 PMC4704271

[21] Vikis H , Sato M , James M , et al. EGFR‐T790M is a rare lung cancer susceptibility allele with enhanced kinase activity. Cancer Res. 2007;67:4665‐4670. 17510392 10.1158/0008-5472.CAN-07-0217 PMC3460269

[22] Hatt M , Laurent B , Fayad H , Jaouen V , Visvikis D , LeRest CC . Tumour functional sphericity from PET images: prognostic value in NSCLC and impact of delineation method. Eur J Nucl Med Mol Imaging. 2018;45:630‐641. 29177871 10.1007/s00259-017-3865-3

[23] Wang SE , Narasanna A , Perez‐Torres M , et al. HER2 kinase domain mutation results in constitutive phosphorylation and activation of HER2 and EGFR and resistance to EGFR tyrosine kinase inhibitors. Cancer Cell. 2006;10:25‐38. 16843263 10.1016/j.ccr.2006.05.023

[24] Wang BX , Ou W , Mao XY , Liu Z , Wu HQ , Wang SY . Impacts of EGFR mutation and EGFR‐TKIs on incidence of brain metastases in advanced non‐squamous NSCLC. Clin Neurol Neurosurg. 2017;160:96‐100. 28704781 10.1016/j.clineuro.2017.06.022

[25] Wang E , Wang Z , Liu S , et al. Polymorphisms of VEGF, TGFbeta1, TGFbetaR2 and conotruncal heart defects in a Chinese population. Mol Biol Rep. 2014;41:1763‐1770. 24443223 10.1007/s11033-014-3025-9

[26] Wang F , Wang S , Wang Z , et al.; Key Laboratory of Carcinogenesis and Translational Research (Ministry of Education) . Phosphorylated EGFR expression may predict outcome of EGFR‐TKIs therapy for the advanced NSCLC patients with wild‐type EGFR. J Exp Clin Cancer Res. 2012;31:65. 22901364 10.1186/1756-9966-31-65 PMC3548765

[27] Jeong EG , Soung YH , Lee JW , et al. ERBB3 kinase domain mutations are rare in lung, breast and colon carcinomas. Int J Cancer. 2006;119:2986‐2987. 16998794 10.1002/ijc.22257

[28] Cappuzzo F , Ligorio C , Toschi L , et al. EGFR and HER2 gene copy number and response to first‐line chemotherapy in patients with advanced non‐small cell lung cancer (NSCLC). J Thorac Oncol. 2007;2:423‐429. 17473658 10.1097/01.JTO.0000268676.79872.9b

[29] Johnson H , Lescarbeau RS , Gutierrez JA , White FM . Phosphotyrosine profiling of NSCLC cells in response to EGF and HGF reveals network specific mediators of invasion. J Proteome Res. 2013;12:1856‐1867. 23438512 10.1021/pr301192t PMC3950895

[30] Rodenhuis S , Slebos RJ . The ras oncogenes in human lung cancer. Am Rev Respir Dis. 1990;142:S27‐30. 2252272 10.1164/ajrccm/142.6_Pt_2.S27

[31] Suzuki Y , Orita M , Shiraishi M , Hayashi K , Sekiya T . Detection of ras gene mutations in human lung cancers by single‐strand conformation polymorphism analysis of polymerase chain reaction products. Oncogene. 1990;5:1037‐1043. 2197591

[32] Rodenhuis S , Slebos RJ , Boot AJ , et al. Incidence and possible clinical significance of K‐ras oncogene activation in adenocarcinoma of the human lung. Cancer Res. 1988;48:5738‐5741. 3048648

[33] Johnson L , Mercer K , Greenbaum D , et al. Somatic activation of the K‐ras oncogene causes early onset lung cancer in mice. Nature. 2001;410:1111‐1116. 11323676 10.1038/35074129

[34] Dankort D , Filenova E , Collado M , Serrano M , Jones K , McMahon M . A new mouse model to explore the initiation, progression, and therapy of BRAFV600E‐induced lung tumors. Genes Dev. 2007;21:379‐384. 17299132 10.1101/gad.1516407 PMC1804325

[35] West KA , Linnoila IR , Belinsky SA , Harris CC , Dennis PA . Tobacco carcinogen‐induced cellular transformation increases activation of the phosphatidylinositol 3'‐kinase/Akt pathway in vitro and in vivo. Cancer Res. 2004;64:446‐451. 14744754 10.1158/0008-5472.can-03-3241

[36] Wislez M , Spencer ML , Izzo JG , et al. Inhibition of mammalian target of rapamycin reverses alveolar epithelial neoplasia induced by oncogenic K‐ras. Cancer Res. 2005;65:3226‐3235. 15833854 10.1158/0008-5472.CAN-04-4420

[37] Heavey S , Cuffe S , Finn S , et al. In pursuit of synergy: an investigation of the PI3K/mTOR/MEK co‐targeted inhibition strategy in NSCLC. Oncotarget. 2016;7:79526‐79543. 27765909 10.18632/oncotarget.12755 PMC5346733

[38] Wan YY , Flavell RA . How diverse–CD4 effector T cells and their functions. J Mol Cell Biol. 2009;1:20‐36. 19482777 10.1093/jmcb/mjp001 PMC2841031

[39] Zhu J , Paul WE . Peripheral CD4+ T‐cell differentiation regulated by networks of cytokines and transcription factors. Immunol Rev. 2010;238:247‐262. 20969597 10.1111/j.1600-065X.2010.00951.x PMC2975272

[40] Wilson NJ , Boniface K , Chan JR , et al. Development, cytokine profile and function of human interleukin 17‐producing helper T cells. Nat Immunol. 2007;8:950‐957. 17676044 10.1038/ni1497

[41] Wilke CM , Kryczek I , Wei S , et al. Th17 cells in cancer: help or hindrance? Carcinogenesis. 2011;32:643‐649. 21304053 10.1093/carcin/bgr019 PMC3086699

[42] Zou W , Restifo NP . T(H)17 cells in tumour immunity and immunotherapy. Nat Rev Immunol. 2010;10:248‐256. 20336152 10.1038/nri2742 PMC3242804

[43] Chang SH , Mirabolfathinejad SG , Katta H , et al. T helper 17 cells play a critical pathogenic role in lung cancer. Proc Natl Acad Sci USA. 2014;111:5664‐5669. 24706787 10.1073/pnas.1319051111 PMC3992670

[44] Shimizu K , Nakata M , Hirami Y , Yukawa T , Maeda A , Tanemoto K . Tumor‐infiltrating Foxp3+ regulatory T cells are correlated with cyclooxygenase‐2 expression and are associated with recurrence in resected non‐small cell lung cancer. J Thorac Oncol. 2010;5:585‐590. 20234320 10.1097/JTO.0b013e3181d60fd7

[45] Duan MC , Zhong XN , Liu GN , Wei JR . The Treg/Th17 paradigm in lung cancer. J Immunol Res. 2014;730380. 24872958 10.1155/2014/730380 PMC4020459

[46] Braumuller H , Wieder T , Brenner E , et al. T‐helper‐1‐cell cytokines drive cancer into senescence. Nature. 2013;494:361‐365. 23376950 10.1038/nature11824

[47] Street SE , Cretney E , Smyth MJ . Perforin and interferon‐gamma activities independently control tumor initiation, growth, and metastasis. Blood. 2001;97:192‐197. 11133760 10.1182/blood.v97.1.192

[48] Haabeth OA , Lorvik KB , Hammarstrom C , et al. Inflammation driven by tumour‐specific Th1 cells protects against B‐cell cancer. Nat Commun. 2011;2:240. 21407206 10.1038/ncomms1239 PMC3072106

[49] Calzascia T , Pellegrini M , Hall H , et al. TNF‐alpha is critical for antitumor but not antiviral T cell immunity in mice. J Clin Invest. 2007;117:3833‐3845. 17992258 10.1172/JCI32567 PMC2066188

[50] Hiraoka O , Satake H , Iguchi S , Matsuda A , Ueda T , Wataya Y . Carbocyclic inosine as a potent anti‐leishmanial agent: the metabolism and selective cytotoxic effects of carbocyclic inosine in promastigotes of Leishmania tropica and Leishmania donovani. Biochem Biophys Res Commun. 1986;134:1114‐1121. 3753868 10.1016/0006-291x(86)90366-9

[51] Neurath MF , Finotto S . The emerging role of T cell cytokines in non‐small cell lung cancer. Cytokine Growth Factor Rev. 2012;23:315‐322. 23022528 10.1016/j.cytogfr.2012.08.009

[52] Gocheva V , Wang HW , Gadea BB , et al. IL‐4 induces cathepsin protease activity in tumor‐associated macrophages to promote cancer growth and invasion. Genes Dev. 2010;24:241‐255. 20080943 10.1101/gad.1874010 PMC2811826

[53] Ochoa CE , Mirabolfathinejad SG , Ruiz VA , et al. Interleukin 6, but not T helper 2 cytokines, promotes lung carcinogenesis. Cancer Prev Res (Phila). 2011;4:51‐64. 21098042 10.1158/1940-6207.CAPR-10-0180 PMC3058282

[54] Hagenbaugh A , Sharma S , Dubinett SM , et al. Altered immune responses in interleukin 10 transgenic mice. J Exp Med. 1997;185:2101‐2110. 9182682 10.1084/jem.185.12.2101 PMC2196349

[55] Terabe M , Park JM , Berzofsky JA . Role of IL‐13 in regulation of anti‐tumor immunity and tumor growth. Cancer Immunol Immunother. 2004;53:79‐85. 14610620 10.1007/s00262-003-0445-0 PMC11034335

[56] de Visser KE , Eichten A , Coussens LM . Paradoxical roles of the immune system during cancer development. Nat Rev Cancer. 2006;6:24‐37. 16397525 10.1038/nrc1782

[57] Middleton GW , Annels NE . Pandha HS . Are we ready to start studies of Th17 cell manipulation as a therapy for cancer? Cancer Immunol Immunother. 2012;61:1‐7. 22086162 10.1007/s00262-011-1151-y PMC11029090

[58] Kryczek I , Banerjee M , Cheng P , et al. Phenotype, distribution, generation, and functional and clinical relevance of Th17 cells in the human tumor environments. Blood. 2009;114:1141‐1149. 19470694 10.1182/blood-2009-03-208249 PMC2723011

[59] Ye J , Zhang Z , Sun L , Fang Y , Xu X , Zhou G . miR‐186 regulates chemo‐sensitivity to paclitaxel via targeting MAPT in non‐small cell lung cancer (NSCLC). Mol Biosyst. 2016;12:3417‐3424. 27714074 10.1039/c6mb00576d

[60] Wang L , Yi T , Kortylewski M , Pardoll DM , Zeng D , Yu H . IL‐17 can promote tumor growth through an IL‐6‐Stat3 signaling pathway. J Exp Med. 2009;206:1457‐1464. 19564351 10.1084/jem.20090207 PMC2715087

[61] Lin WW , Karin M . A cytokine‐mediated link between innate immunity, inflammation, and cancer. J Clin Invest. 2007;117:1175‐1183. 17476347 10.1172/JCI31537 PMC1857251

[62] Li L , Chao QG , Ping LZ , et al. The prevalence of FOXP3+ regulatory T‐cells in peripheral blood of patients with NSCLC. Cancer Biother Radiopharm. 2009;24:357‐367. 19538059 10.1089/cbr.2008.0612

[63] Sharma S , Stolina M , Lin Y , et al. T cell‐derived IL‐10 promotes lung cancer growth by suppressing both T cell and APC function. J Immunol. 1999;163:5020‐5028. 10528207

[64] Beyer F , Buerke B , Gerss J , et al. Prediction of lymph node metastases in NSCLC. three dimensional anatomical parameters do not substitute FDG‐PET‐CT. Nuklearmedizin. 2010;49:41‐48. quiz N41. 20087533 10.3413/nukmed-0277

[65] Hasegawa Y , Takanashi S , Kanehira Y , Tsushima T , Imai T , Okumura K . Transforming growth factor‐beta1 level correlates with angiogenesis, tumor progression, and prognosis in patients with nonsmall cell lung carcinoma. Cancer. 2001;91:964‐971. 11251948

[66] Roberts AB , Wakefield LM . The two faces of transforming growth factor beta in carcinogenesis. Proc Natl Acad Sci USA. 2003;100:8621‐8623. 12861075 10.1073/pnas.1633291100 PMC166359

[67] Schneberger D , Aharonson‐Raz K , Singh B . Monocyte and macrophage heterogeneity and Toll‐like receptors in the lung. Cell Tissue Res. 2011;343:97‐106. 20824285 10.1007/s00441-010-1032-2

[68] Sica A , Allavena P , Mantovani A . Cancer related inflammation: the macrophage connection. Cancer Lett. 2008;267:204‐215. 18448242 10.1016/j.canlet.2008.03.028

[69] Ma J , Liu L , Che G , Yu N , Dai F , You Z . The M1 form of tumor‐associated macrophages in non‐small cell lung cancer is positively associated with survival time. BMC Cancer. 2010;10:112. 20338029 10.1186/1471-2407-10-112 PMC2851690

[70] Guermonprez P , Valladeau J , Zitvogel L , Thery C , Amigorena S . Antigen presentation and T cell stimulation by dendritic cells. Annu Rev Immunol. 2002;20:621‐667. 11861614 10.1146/annurev.immunol.20.100301.064828

[71] Perrot I , Blanchard D , Freymond N , et al. Dendritic cells infiltrating human non‐small cell lung cancer are blocked at immature stage. J Immunol. 2007;178:2763‐2769. 17312119 10.4049/jimmunol.178.5.2763

[72] Vivier E , Ugolini S , Blaise D . Chabannon C , Brossay L . Targeting natural killer cells and natural killer T cells in cancer. Nat Rev Immunol. 2012;12:239‐252. 22437937 10.1038/nri3174 PMC5161343

[73] Culley FJ . Natural killer cells in infection and inflammation of the lung. Immunology. 2009;128:151‐163. 19740372 10.1111/j.1365-2567.2009.03167.x PMC2767305

[74] Platonova S , Cherfils‐Vicini J , Damotte D , et al. Profound coordinated alterations of intratumoral NK cell phenotype and function in lung carcinoma. Cancer Res. 2011;71:5412‐5422. 21708957 10.1158/0008-5472.CAN-10-4179

[75] Hodge G , Barnawi J , Jurisevic C , et al. Lung cancer is associated with decreased expression of perforin, granzyme B and interferon (IFN)‐gamma by infiltrating lung tissue T cells, natural killer (NK) T‐like and NK cells. Clin Exp Immunol. 2014;178:79‐85. 24894428 10.1111/cei.12392 PMC4360197

[76] Carrega P , Bonaccorsi I , Di Carlo E , et al. CD56(bright)perforin(low) noncytotoxic human NK cells are abundant in both healthy and neoplastic solid tissues and recirculate to secondary lymphoid organs via afferent lymph. J Immunol. 192:3805‐3815. 24646734 10.4049/jimmunol.1301889

[77] Bruno A , Focaccetti C , Pagani A , et al. The proangiogenic phenotype of natural killer cells in patients with non‐small cell lung cancer. Neoplasia. 2013;15:133‐142. 23441128 10.1593/neo.121758 PMC3579316

[78] Esendagli G , Bruderek K , Goldmann T , et al. Malignant and non‐malignant lung tissue areas are differentially populated by natural killer cells and regulatory T cells in non‐small cell lung cancer. Lung Cancer. 2008;59:32‐40. 17825949 10.1016/j.lungcan.2007.07.022

[79] Schneider BJ , Kalemkerian GP , Kraut MJ , et al. Phase II study of celecoxib and docetaxel in non‐small cell lung cancer (NSCLC) patients with progression after platinum‐based therapy. J Thorac Oncol. 2008;3:1454‐1459. 19057272 10.1097/JTO.0b013e31818de1d2 PMC3771331

[80] Liu L , Yao J , Ding Q , Huang S . CD4+CD25 high regulatory cells in peripheral blood of NSCLC patients. J Huazhong Univ Sci Technol Med Sci. 2006;26:548‐551. 17219964 10.1007/s11596-006-0516-5

[81] Chmielewski M , Hombach AA . Abken H . Antigen‐specific T‐cell activation independently of the MHC: chimeric antigen receptor‐redirected T cells. Front Immunol. 2013;4:371. 24273543 10.3389/fimmu.2013.00371 PMC3822734

[82] Postmus PE , Kerr KM , Oudkerk M , et al.; ESMO Guidelines Committee . Early and locally advanced non‐small‐cell lung cancer (NSCLC): ESMO Clinical Practice Guidelines for diagnosis, treatment and follow‐up. Ann Oncol. 2017;28:iv1‐iv21. 28881918 10.1093/annonc/mdx222

[83] Pignon J‐P , Tribodet H , Scagliotti GV , et al. Lung adjuvant cisplatin evaluation: a pooled analysis by the LACE Collaborative Group. J Clin Oncol. 2008;26:3552‐3559. 18506026 10.1200/JCO.2007.13.9030

[84] Thunnissen E , Kerr KM , Herth FJ , et al. The challenge of NSCLC diagnosis and predictive analysis on small samples. practical approach of a working group. Lung Cancer. 2012;76:1‐18. 22138001 10.1016/j.lungcan.2011.10.017

[85] Alamgeer M , Ganju V , Watkins DN . Novel therapeutic targets in non‐small cell lung cancer. Curr Opin Pharmacol. 2013;13:394‐401. 23608109 10.1016/j.coph.2013.03.010

[86] Savas P , Hughes B , Solomon B . Targeted therapy in lung cancer: IPASS and beyond, keeping abreast of the explosion of targeted therapies for lung cancer. J Thorac Dis. 2013;5(Suppl 5):S579‐S592. 24163750 10.3978/j.issn.2072-1439.2013.08.52 PMC3804878

[87] Burgess AW , Cho H‐S , Eigenbrot C , et al. An open‐and‐shut case? recent insights into the activation of EGF/ErbB receptors. Mol Cell. 2003;12:541‐552. 14527402 10.1016/s1097-2765(03)00350-2

[88] Kimura H , Sakai K , Arao T , Shimoyama T , Tamura T , Nishio K . Antibody‐dependent cellular cytotoxicity of cetuximab against tumor cells with wild‐type or mutant epidermal growth factor receptor. Cancer Sci. 2007;98:1275‐1280. 17498200 10.1111/j.1349-7006.2007.00510.x PMC11159318

[89] Ennis BW , Lippman ME , Dickson RB . The EGF receptor system as a target for antitumor therapy. Cancer Invest. 1991;9:553‐562. 1933488 10.3109/07357909109018953

[90] Ciardiello F . Epidermal growth factor receptor tyrosine kinase inhibitors as anticancer agents. Drugs. 2000;60(Suppl 1):25‐32. discussion 41‐22. 11129169 10.2165/00003495-200060001-00003

[91] Mok TS , Wu Y‐L , Thongprasert S , et al. Gefitinib or carboplatin‐paclitaxel in pulmonary adenocarcinoma. N Engl J Med. 2009;361:947‐957. 19692680 10.1056/NEJMoa0810699

[92] Inoue A , Kobayashi K , Maemondo M , et al. Updated overall survival results from a randomized phase III trial comparing gefitinib with carboplatin‐paclitaxel for chemo‐naive non‐small cell lung cancer with sensitive EGFR gene mutations (NEJ002). Ann Oncol. 2013;24:54‐59. 22967997 10.1093/annonc/mds214

[93] Maemondo M , Inoue A , Kobayashi K , et al. Gefitinib or chemotherapy for non‐small‐cell lung cancer with mutated EGFR. N Engl J Med. 2010;362:2380‐2388. 20573926 10.1056/NEJMoa0909530

[94] Fukuoka M , Yano S , Giaccone G , et al. Multi‐institutional randomized phase II trial of gefitinib for previously treated patients with advanced non‐small‐cell lung cancer (The IDEAL 1 Trial) [corrected]. J Clin Oncol. 2003;21:2237‐2246. 12748244 10.1200/JCO.2003.10.038

[95] Arteaga CL , Johnson DH . Tyrosine kinase inhibitors‐ZD1839 (Iressa). Curr Opin Oncol. 2001;13:491‐498. 11673690 10.1097/00001622-200111000-00012

[96] Moyer JD , Barbacci EG , Iwata KK , et al. Induction of apoptosis and cell cycle arrest by CP‐358,774, an inhibitor of epidermal growth factor receptor tyrosine kinase. Cancer Res. 1997;57:4838‐4848. 9354447

[97] Ranson M , Reck M , Anthoney A , et al. Erlotinib in combination with pemetrexed for patients with advanced non‐small‐cell lung cancer (NSCLC): a phase I dose‐finding study. Ann Oncol. 2010;21:2233‐2239. 20444843 10.1093/annonc/mdq246

[98] Miller VA , Hirsh V , Cadranel J , et al. Afatinib versus placebo for patients with advanced, metastatic non‐small‐cell lung cancer after failure of erlotinib, gefitinib, or both, and one or two lines of chemotherapy (LUX‐Lung 1): a phase 2b/3 randomised trial. Lancet Oncol. 2012;13:528‐538. 22452896 10.1016/S1470-2045(12)70087-6

[99] Reckamp KL , Giaccone G , Camidge DR , et al. A phase 2 trial of dacomitinib (PF‐00299804), an oral, irreversible pan‐HER (human epidermal growth factor receptor) inhibitor, in patients with advanced non‐small cell lung cancer after failure of prior chemotherapy and erlotinib. Cancer. 2014;120:1145‐1154. 24501009 10.1002/cncr.28561 PMC4164026

[100] Regales L , Gong Y , Shen R , et al. Dual targeting of EGFR can overcome a major drug resistance mutation in mouse models of EGFR mutant lung cancer. J Clin Invest. 2009;119:3000‐3010. 19759520 10.1172/JCI38746 PMC2752070

[101] Janjigian YY , Smit EF , Groen HJ , et al. Dual inhibition of EGFR with afatinib and cetuximab in kinase inhibitor‐resistant EGFR‐mutant lung cancer with and without T790M mutations. Cancer Discov. 2014;4:1036‐1045. 25074459 10.1158/2159-8290.CD-14-0326 PMC4155006

[102] Planchard D , Loriot Y , Andre F , et al. EGFR‐independent mechanisms of acquired resistance to AZD9291 in EGFR T790M‐positive NSCLC patients. Ann Oncol. 2015;26: 2073‐2078. 26269204 10.1093/annonc/mdv319

[103] Park K , Lee JS , Han JY , et al. 1300: Efficacy and safety of BI 1482694 (HM61713), an EGFR mutant‐specific inhibitor, in T790M‐positive NSCLC at the recommended phase II dose. J Thorac Oncol. 2016;11:S113.

[104] Tan CS , Cho BC , Soo RA . Treatment options for EGFR mutant NSCLC with CNS involvement‐Can patients BLOOM with the use of next generation EGFR TKIs? Lung Cancer. 2017;108:29‐37. 28625644 10.1016/j.lungcan.2017.02.012

[105] Ghafoor Q , Baijal S , Taniere P , O'Sullivan B , Evans M , Middleton G . Epidermal growth factor receptor (EGFR) kinase inhibitors and non‐small cell lung cancer (NSCLC)—advances in molecular diagnostic techniques to facilitate targeted therapy. Pathol Oncol Res. 2018;24:723‐731. 29270776 10.1007/s12253-017-0377-1

[106] Ostrem JM , Peters U , Sos ML , Wells JA . Shokat KM K‐Ras (G12C) inhibitors allosterically control GTP affinity and effector interactions. Nature. 2013;503:548‐551. 24256730 10.1038/nature12796 PMC4274051

[107] Sousa SF , Fernandes PA , Ramos MJ . Farnesyltransferase inhibitors: a detailed chemical view on an elusive biological problem. Curr Med Chem. 2008;15:1478‐1492. 18537624 10.2174/092986708784638825

[108] Tsimberidou AM , Rudek MA , Hong D , et al. Phase 1 first‐in‐human clinical study of S‐trans, trans‐farnesylthiosalicylic acid (salirasib) in patients with solid tumors. Cancer Chemother Pharmacol. 2010;65:235‐241. 19484470 10.1007/s00280-009-1027-4

[109] Riely GJ . The use of first‐generation tyrosine kinase inhibitors in patients with NSCLC and somatic EGFR mutations. Lung Cancer. 2008;60(Suppl 2):S19‐22. 18513580 10.1016/S0169-5002(08)70101-6

[110] Hunter JC , Gurbani D , Ficarro SB , et al. In situ selectivity profiling and crystal structure of SML‐8‐73‐1, an active site inhibitor of oncogenic K‐Ras G12C. Proc Natl Acad Sci USA. 2014;111:8895‐8900. 24889603 10.1073/pnas.1404639111 PMC4066474

[111] Janes MR , Zhang J , Li LS , et al. Targeting KRAS mutant cancers with a covalent G12C‐specific inhibitor. Cell. 2018;172:578‐589. e517 29373830 10.1016/j.cell.2018.01.006

[112] Garon EB . The race for combined checkpoint inhibition in NSCLC. Lancet Oncol. 2016;17 :259‐260. 26858123 10.1016/S1470-2045(15)00580-X

[113] Janne PA , Shaw AT , Pereira JR , et al. Selumetinib plus docetaxel for KRAS‐mutant advanced non‐small‐cell lung cancer: a randomised, multicentre, placebo‐controlled, phase 2 study. Lancet Oncol. 2013;14:38‐47. 23200175 10.1016/S1470-2045(12)70489-8

[114] Tolcher AW , Patnaik A , Papadopoulos KP , et al. Phase I study of the MEK inhibitor trametinib in combination with the AKT inhibitor afuresertib in patients with solid tumors and multiple myeloma. Cancer Chemother Pharmacol. 2015;75: 183‐189. 25417902 10.1007/s00280-014-2615-5

[115] Sherr CJ , Beach D , Shapiro GI . Targeting CDK4 and CDK6: from discovery to therapy. Cancer Discov. 2016;6:353‐367. 26658964 10.1158/2159-8290.CD-15-0894 PMC4821753

[116] Goldman JW , Mazieres J , Barlesi F , et al. A randomized phase 3 study of abemaciclib versus erlotinib in previously treated patients with stage IV NSCLC with KRAS mutation: JUNIPER. J Clin Oncol. 2018;36.

[117] Wilhelm SM , Adnane L , Newell P , Villanueva A , Llovet JM , Lynch M . Preclinical overview of sorafenib, a multikinase inhibitor that targets both Raf and VEGF and PDGF receptor tyrosine kinase signaling. Mol Cancer Ther. 2008;7:3129‐3140. 18852116 10.1158/1535-7163.MCT-08-0013 PMC12261297

[118] Landi L . Cappuzzo F . Targeting MET in NSCLC: looking for a needle in a haystack. Transl Lung Cancer Res. 2014;3:389‐391. 25806329 10.3978/j.issn.2218-6751.2014.11.05 PMC4367673

[119] Korman AJ , Peggs KS , Allison JP . Checkpoint blockade in cancer immunotherapy. Adv Immunol. 2006;90:297‐339. 16730267 10.1016/S0065-2776(06)90008-X PMC1951510

[120] Pardoll DM . The blockade of immune checkpoints in cancer immunotherapy. Nat Rev Cancer. 2012;12:252‐264. 22437870 10.1038/nrc3239 PMC4856023

[121] Zou W , Chen L . Inhibitory B7‐family molecules in the tumour microenvironment. Nat Rev Immunol. 2008;8:467‐477. 18500231 10.1038/nri2326

[122] Rudd CE , Taylor A , Schneider H . CD28 and CTLA‐4 coreceptor expression and signal transduction. Immunol Rev. 2009;229:12‐26. 19426212 10.1111/j.1600-065X.2009.00770.x PMC4186963

[123] Lynch TJ , Bondarenko I , Luft A , et al. Ipilimumab in combination with paclitaxel and carboplatin as first‐line treatment in stage IIIB/IV non‐small‐cell lung cancer: results from a randomized, double‐blind, multicenter phase II study. J Clin Oncol. 2012;30:2046‐2054. 22547592 10.1200/JCO.2011.38.4032

[124] Ribas A , Hanson Dc , Noe Da , et al. Tremelimumab (CP‐675,206), a cytotoxic T lymphocyte associated antigen 4 blocking monoclonal antibody in clinical development for patients with cancer. Oncologist. 2007;12:873‐883. 17673618 10.1634/theoncologist.12-7-873

[125] Brahmer JR , Rodriguez‐Abreu D , Robinson AG , et al. Health‐related quality‐of‐life results for pembrolizumab versus chemotherapy in advanced, PD‐L1‐positive NSCLC (KEYNOTE‐024): a multicentre, international, randomised, open‐label phase 3 trial. Lancet Oncol. 2017;18:1600‐1609. 29129441 10.1016/S1470-2045(17)30690-3

[126] Sundar R , Cho BC , Brahmer JR , Soo RA . Nivolumab in NSCLC: latest evidence and clinical potential. Ther Adv Med Oncol. 2015;7:85‐96. 25755681 10.1177/1758834014567470 PMC4346216

[127] Brahmer JR , Tykodi SS , Chow LQ , et al. Safety and activity of anti‐PD‐L1 antibody in patients with advanced cancer. N Engl J Med. 2012;366:2455‐2465. 22658128 10.1056/NEJMoa1200694 PMC3563263

[128] Herbst RS , De Marinis F , Jassem J , et al. 56: IMpower110: phase III trial comparing 1L atezolizumab with chemotherapy in PD‐L1‐selected chemotherapy‐naive NSCLC patients: topic: medical oncology. J Thorac Oncol. 2016;11:S304‐S305.

[129] Drake CG , Lipson EJ , Brahmer JR . Breathing new life into immunotherapy: review of melanoma, lung and kidney cancer. Nat Rev Clin Oncol. 2014;11:24‐37. 24247168 10.1038/nrclinonc.2013.208 PMC4086654

[130] Cuppens K , Vansteenkiste J . Vaccination therapy for non‐small‐cell lung cancer. Curr Opin Oncol. 2014;26:165‐170. 24441502 10.1097/CCO.0000000000000052

[131] Giaccone GBL , Nemunaitis J . A phase III study of belagenpumatucel‐L therapeutic tumor cell vaccine for non–small cell lung cancer. Proceedings of the European Cancer Congress Abstract nr 7081. 2013.

[132] Samuel J , Budzynski WA , Reddish MA , et al. Immunogenicity and antitumor activity of a liposomal MUC1 peptide‐based vaccine. Int J Cancer. 1998;75:295‐302. 9462722 10.1002/(sici)1097-0215(19980119)75:2<295::aid-ijc20>3.0.co;2-b

[133] Butts C , Socinski MA , Mitchell PL , et al. Tecemotide (L‐BLP25) versus placebo after chemoradiotherapy for stage III non‐small‐cell lung cancer (START): a randomised, double‐blind, phase 3 trial. Lancet Oncol. 2014;15:59‐68. 24331154 10.1016/S1470-2045(13)70510-2

[134] Jang SJ , Soria JC , Wang L , et al. Activation of melanoma antigen tumor antigens occurs early in lung carcinogenesis. Cancer Res. 2001;61:7959‐7963. 11691819

[135] Vansteenkiste J , Zielinski M , Linder A , et al. Adjuvant MAGE‐A3 immunotherapy in resected non‐small‐cell lung cancer: phase II randomized study results. J Clin Oncol. 2013;31:2396‐2403. 23715567 10.1200/JCO.2012.43.7103

[136] Ulloa‐Montoya F , Louahed J , Dizier B , et al. Predictive gene signature in MAGE‐A3 antigen‐specific cancer immunotherapy. J Clin Oncol. 2013;31:2388‐2395. 23715562 10.1200/JCO.2012.44.3762

[137] Quoix E . Stage IV NSCLC. second‐line therapy for metastatic non‐small cell lung cancer. Rev Mal Respir. 2008;25:3S113‐118. 18971835

[138] Baron MG , Giron CG , Zamora P , et al. Non‐small‐cell lung cancer (NSCLC): chemotherapy in advanced disease. Our experience in ten years. Am J Clin Oncol. 1992;15:23‐28. 1312769 10.1097/00000421-199202000-00005

[139] Garcia‐Campelo R , Bernabe R , Cobo M , et al. SEOM clinical guidelines for the treatment of non‐small cell lung cancer (NSCLC). Clin Transl Oncol. 2015;17:1020‐1029. 26691657 10.1007/s12094-015-1455-z PMC4689744

[140] Noda K , Tanaka K , Yamada A , Ogata J , Tanaka H , Shoyama Y . Simple assay for antitumour immunoactive glycoprotein derived from Chlorella vulgaris strain CK22 using ELISA. Phytother Res. 2002;16:581‐585. 12237820 10.1002/ptr.1021

[141] Furusawa E , Furusawa S . Anticancer potential of Viva‐Natural, a dietary seaweed extract, on Lewis lung carcinoma in comparison with chemical immunomodulators and on cyclosporine‐accelerated AKR leukemia. Oncology. 1989;46:343‐348. 2476696 10.1159/000226746

[142] Furusawa E , Furusawa S . Effect of pretazettine and viva‐natural, a dietary seaweed extract, on spontaneous AKR leukemia in comparison with standard drugs. Oncology. 1988;45:180‐186. 3368194 10.1159/000226558

[143] Kyo E , Uda N , Kakimoto M , et al. Anti‐allergic effects of aged garlic extract. Phytomedicine. 1997;4:335‐340. 23195584 10.1016/S0944-7113(97)80043-8

[144] Kyo E , Uda N , Suzuki A , et al. Immunomodulation and antitumor activities of Aged Garlic Extract. Phytomedicine. 1998;5:259‐267. 23195897 10.1016/S0944-7113(98)80064-0

[145] Mishra LC , Singh BB , Dagenais S . Scientific basis for the therapeutic use of Withania somnifera (ashwagandha): a review. Altern Med Rev. 2000;5:334‐346. 10956379

[146] Brown MD . Green tea (Camellia sinensis) extract and its possible role in the prevention of cancer. Altern Med Rev. 1999;4:360‐370. 10559550

[147] Yuan JM . Cancer prevention by green tea: evidence from epidemiologic studies. Am J Clin Nutr. 2013;98:1676S‐1681S. 24172305 10.3945/ajcn.113.058271 PMC3831544

[148] Goyal S , Jamal S , Shanker A , Grover A . Structural investigations of T854A mutation in EGFR and identification of novel inhibitors using structure activity relationships. BMC Genomics. 2015;16(Suppl 5):S8. 10.1186/1471-2164-16-S5-S8 PMC4460657 26041145

[149] Xiang M , Lei K , Fan W , et al. In silico identification of EGFR‐T790M inhibitors with novel scaffolds: start with extraction of common features. Drug Des Devel Ther. 2013;7:789‐839. 10.2147/DDDT.S41305 PMC3748928 23990708

[150] Lu J , Chen L , Yin J , et al. Identification of new candidate drugs for lung cancer using chemical‐chemical interactions, chemical‐protein interactions and a K‐means clustering algorithm. J Biomol Struct Dyn. 2016;34: 906‐917. 26849843 10.1080/07391102.2015.1060161

